# Motor Mapping of the Brain: Taniguchi Versus Penfield Method

**DOI:** 10.7759/cureus.24901

**Published:** 2022-05-11

**Authors:** Faisal R Jahangiri, Marie Liang, Shabab S Kabir, Oly Khowash

**Affiliations:** 1 Neurology, AINeuroCare Academy, Dallas, USA; 2 Neurophysiology, Global Innervation LLC, Dallas, USA; 3 Intraoperative Neuromonitoring Program, Labouré College of Healthcare, Milton, USA; 4 School of Behavioral and Brain Sciences, University of Texas at Dallas, Richardson, USA; 5 Neuroscience, School of Behavioral and Brain Sciences, University of Texas at Dallas, Richardson, USA

**Keywords:** motor mapping, penfield, taniguchi, cortical mapping, neurophysiology, neuromonitoring, ionm

## Abstract

Intraoperative neurophysiological monitoring (IONM) techniques continue to prove useful as an adjunct in select surgeries for reducing the incidence of various postoperative deficits in motor function through the monitoring of motor evoked potentials (MEPs). The Penfield and Taniguchi methods of direct electrical cortical stimulation (DECS) stand in contrast to each other. Penfield’s method uses lower-frequency stimulation over a longer duration, while Taniguchi’s method uses a relatively higher frequency over a short duration. DECS motor mapping is considered suitable for tumor resections, aneurysm surgeries, arteriovenous malformation, and epilepsy surgeries. While subcortical motor mapping works efficiently with both methods, it aligns with Taniguchi’s method more effectively. Taniguchi’s method has a lower risk of seizures relative to Penfield’s method. While only cortical neurons are excited in Penfield’s stimulation technique, Taniguchi’s technique excites the whole corticospinal tract (CST), so it can be used for mapping in a stand-alone fashion. The Penfield technique remains the method of choice for language mapping. In all motor mapping, Train-of-Four (TOF) stimulation during the surgical procedure ensures that the patient’s muscles are not unduly relaxed.

## Introduction

As target eloquent tissue may be located over a wide range of motor areas [1], procedures involving aneurysms, arteriovenous malformations, epilepsy, and tumors can indirectly threaten the integrity of blood vessels, resulting in dysfunctions in the corticospinal tract (CST) via complications such as subarachnoid hemorrhages and ischemic necrosis [2]. At the same time, tumors may also introduce more instability by causing shifts in regions and making their identification remarkably unreliable [3]. In cases involving such adverse outcomes, injuries sustained intraoperatively invariably translate into deficits severely hampering the patient’s quality of life, such as pareses, paralyzes, coordination issues across gross and fine realms, sluggishness, and pain.

Neuromonitoring can be utilized intraoperatively to localize these regions of interest and reduce the likelihood of deficits emerging. Monitoring yields benefits in the outcome of the surgeries, as mentioned earlier, in terms of shortening the duration, minimizing the severity, intraoperative identification, reversal, and outright circumvention of postoperative deficits. Using a multimodal approach including sensory and motor mapping protocols, electroencephalography (EEG), electrocorticography (ECoG), electromyography (EMG), and Train-of-Four (TOF) optimizes sensitivity and specificity for predicting postoperative deficits [4], such that accidental damage resulting in new deficits following surgery may be circumvented or reversed intraoperatively, should the opportunity and need to arise. Direct electrical cortical stimulation (DECS), reinforced with ECoG and EEG information, can thus be considered vital to preserving healthy corticospinal tract functioning. While direct cortical electrical stimulation protocols have been used for over a century and have seen much refinement, two chief composites using a few substantially different parameters have surfaced. Penfield created a method for motor mapping in 1937, which was adapted in 1991 for use in patients under general anesthesia. In 1993, Taniguchi modified the Penfield method. The present work seeks to evaluate both techniques in terms of their contrasts in procedures that they may or may not be appropriate for, the risks they present, and the benefits in understanding they provide.

## Technical report

Patient selection

Patients undergoing surgeries involving eloquent tissue where the integrity of the motor cortex is at risk or close to the surgical site are ideal candidates for DECS. This includes brain tumor resection [3], focal resections for temporal lobe epilepsy surgeries [5], craniotomies, angioma resections [6], and intracerebral aneurysm surgeries [2].

Anesthesia

Total intravenous anesthesia (TIVA) is the current standard anesthesia protocol in various surgeries and is recommended for use with the recording of motor evoked potentials (MEPs). MEPs are more susceptible to halogenated anesthesia, which can alter the amplitude and latency of responses, and, thus, should be avoided if possible. A TIVA protocol is recommended with propofol and an infusion of remifentanil or fentanyl. Muscle relaxants should be avoided due to their interference with MEP and DECS recordings; however, rocuronium or succinylcholine are muscle relaxants that can be given at intubation and wear off within five minutes or 30 minutes, respectively. If necessary, they can also be reversed by a dose of sugammadex [3]. For language mapping, an asleep-awake-asleep (AAA) protocol or laryngeal mask anesthesia (LMA) should be used [6].

TOF should be recorded from the abductor hallucis and extensor hallucis brevis muscles to ensure no neuromuscular junction blockage during the MEP and DECS recordings [7]. Anti-epileptics should be administered to avoid the likelihood of seizures caused by stimulation, except in focal epilepsy resection procedures where monitoring of seizure activity is necessary.

Sensory mapping of the cortex

Recording for cortical somatosensory evoked potentials (SSEPs) is performed using grid electrodes placed on the exposed cortex, with each grid contact referencing FPz as a channel (e.g., G1-FPz and G2-FPz). The median and ulnar nerves are used for the upper extremity SSEPs, and the posterior tibial and peroneal nerves are stimulated for the lower extremity SSEPs. Recordings are taken by stimulating the contralateral nerves. The bandpass filter should be set to 30-3,000 Hertz (Hz) and have sensitivity to 1-5 microvolts (µV)/division. The sweep should be 10 milliseconds (ms)/division with an input gain of 50 µV/division, pulse width of 0.3 ms, repetition rate of 2.79, and stimulation intensity of 25-30 milliamperes (mA) for the median nerve, 15-20 mA for the ulnar nerve, and 45-100 mA for the tibial nerve [8].

**Figure 1:**
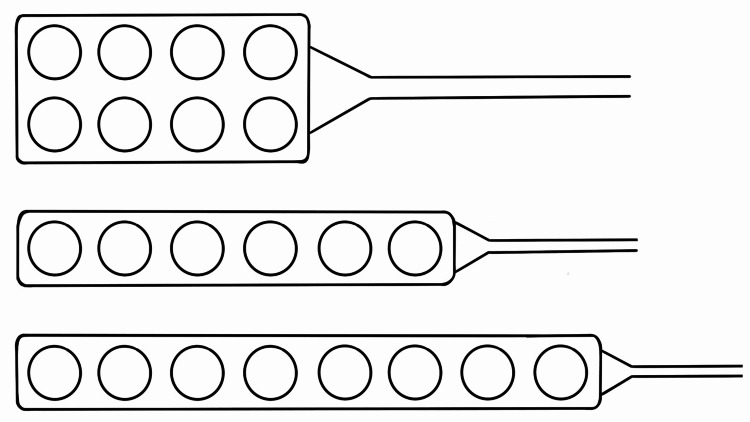
Grid electrodes used for direct cortical stimulation (DCS) and recording during cortical mapping procedures From top to bottom: a 2 × 4 contacts grid electrode, a 1 × 6 contacts grid electrode, and a 1 × 8 contacts grid electrode

Baseline SSEP recordings should be collected from electrodes placed on the scalp according to the international 10-20 system at CP3, CPz, CP4, FPz, cervical vertebra five (CV5), Erb’s point, and the popliteal fossa (PF) before the dura is opened. The localization of the central sulcus on the exposed cortex is performed by placing cortical grid electrodes directly on the cortical surface and eliciting the strongest N20-P30/P22-N30 or N20-P25-P30/P22-P25-N30 responses for the SSEP phase reversal (Figure 1) [9].

Motor mapping of the cortex and electromyography

Compound muscle action potential (CMAP) for electromyography (EMG) should be recorded from muscles of interest contralateral to the stimulation site using subdermal needle electrodes. In the case of midline tumors, recordings are done in bilateral muscles. The recommended muscles for recording motor evoked potentials (MEPs) are the orbicularis oris and tongue (for non-awake surgeries) for the face, deltoid for the shoulder, brachioradialis and flexor carpi ulnaris for the forearm, abductor pollicis brevis, abductor digiti minimi, and first dorsal interosseous muscles for the hand, and tibialis anterior and abductor hallucis for the lower extremities (Table 1) [10].

The recording sensitivity should be 50-200 µV/division, with a bandpass filter set from 10 to 5,000 Hz and a sweep of 100 ms [2,9]. Cortical areas with positive mapping should be marked with the corresponding muscle to map the homunculus of the motor cortex. Motor evoked potential or recordings can be conducted from the same muscles.

**Table 1:** Recommended muscles for recording motor evoked potentials and electromyography Motor evoked potential and electromyography recordings can be conducted from the same muscles.

Recommended muscles	Body area monitored
Orbicularis oris, tongue	Face
Deltoid, biceps brachii	Shoulder
Brachioradialis, flexor carpi ulnaris	Forearm
Abductor pollicis brevis, abductor digiti minimi, first dorsal interosseous	Hand
Quadriceps, tibialis anterior, gastrocnemius	Leg
Abductor hallucis	Foot

Penfield method

**Figure 2:**
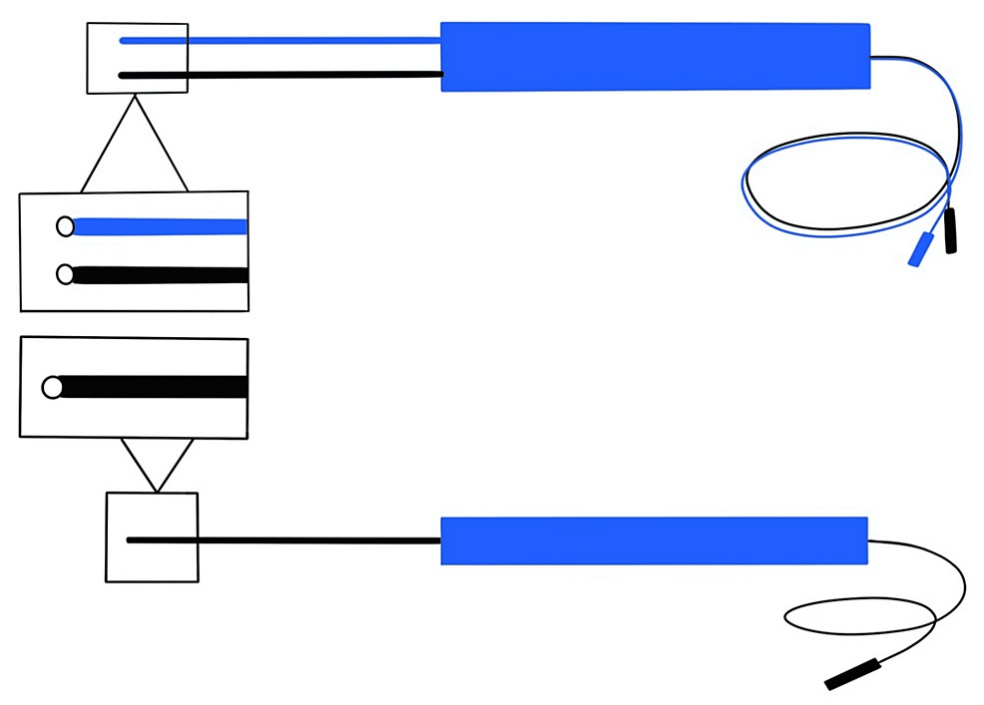
Handheld ball tip stimulation probes used for motor mapping Top: bipolar stimulation probe used in the Penfield method; bottom: monopolar stimulation probe used in the Taniguchi method Anodal stimulation is used for cortical motor mapping, and cathodal stimulation is used for subcortical motor mapping.

**Figure 3:**
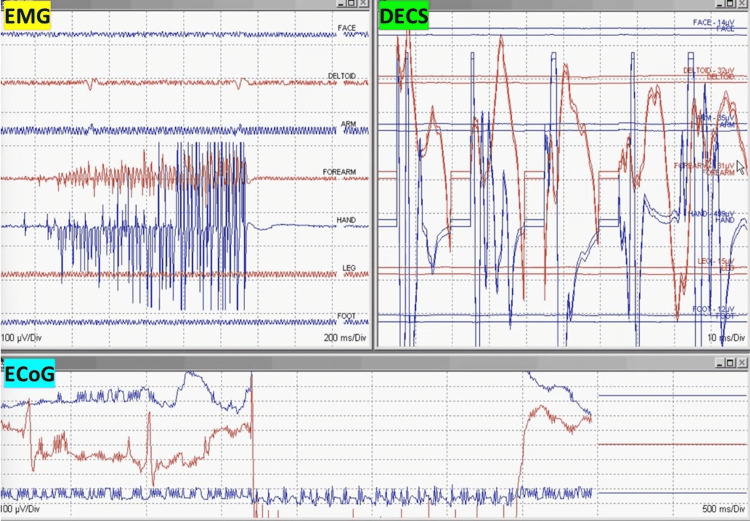
Penfield motor mapping method Penfield 50 Hz motor mapping evoked responses after bipolar handheld stimulation. Multiple responses are recorded in the forearm and hand muscles. Face: orbicularis oris; deltoid, arm: biceps brachii; forearm: brachioradialis/flexor carpi ulnaris; hand: abductor pollicis brevis/abductor digiti minimi; leg: tibialis anterior; foot: abductor hallucis EMG: electromyography; DECS: Penfield direct electrical cortical stimulation; ECoG: electrocorticography

The Penfield method uses a handheld bipolar ball tip probe for direct cortical stimulation (DCS) with a 50 or 60 Hz stimulation frequency (Figure 2) and a train of monophasic pulses with each pulse width between 200 and 1,000 microseconds (µs). The direct cortical stimulation is applied for 2-5 seconds. Stimulation intensity is increased stepwise from 1 mA until a response is generated in the recording muscles until an after discharge (AD) occurs or until a maximum of 20 mA intensity is reached (Figure 3). Electrocorticography (ECoG) recordings are performed with DCS to identify the presence of any after discharges (AD) due to stimulation. Iced saline (4°C) is applied immediately if an AD is noted to prevent the development of a seizure [2]. The Penfield method is preferred for surgeries involving language mapping due to its nature of longer stimulus duration.

Taniguchi method

**Figure 4:**
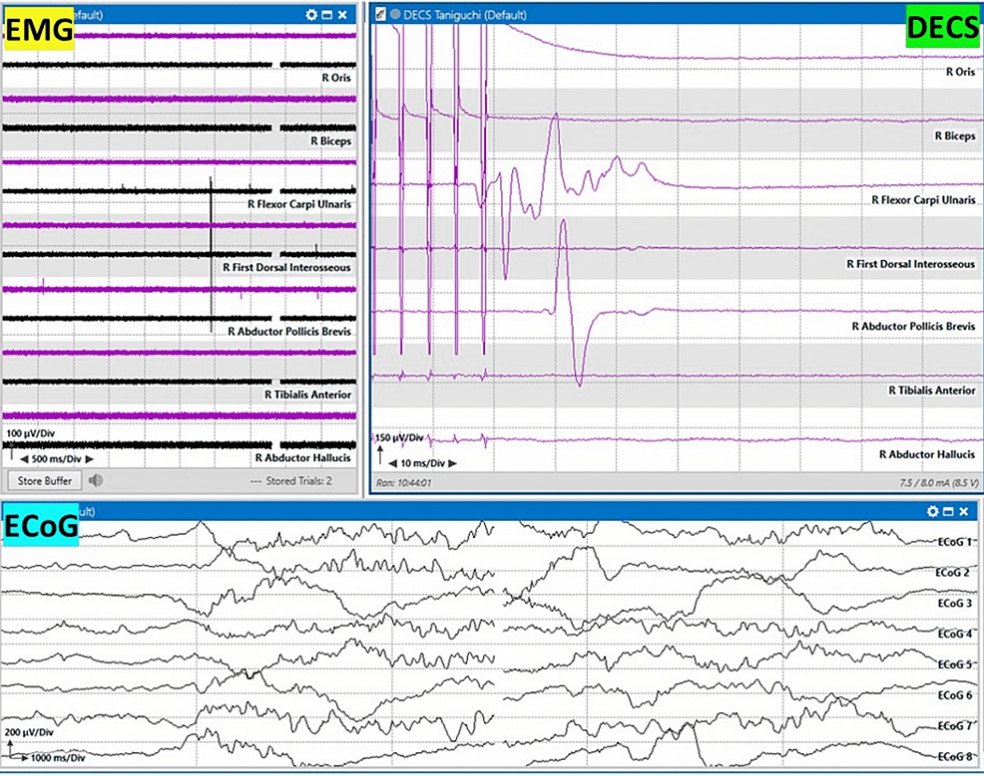
Taniguchi motor mapping method Taniguchi 320 Hz motor mapping evoked responses after monopolar handheld stimulation. Responses are recorded in the forearm (flexor carpi ulnaris) and hand (abductor pollicis brevis) muscles. Oris: orbicularis oris; biceps: biceps brachii; flexor carpi ulnaris, first dorsal interosseous, abductor pollicis brevis, tibialis anterior, and abductor hallucis muscles EMG: electromyography; DECS: Taniguchi direct electrical cortical stimulation; ECoG: electrocorticography

**Figure 5:**
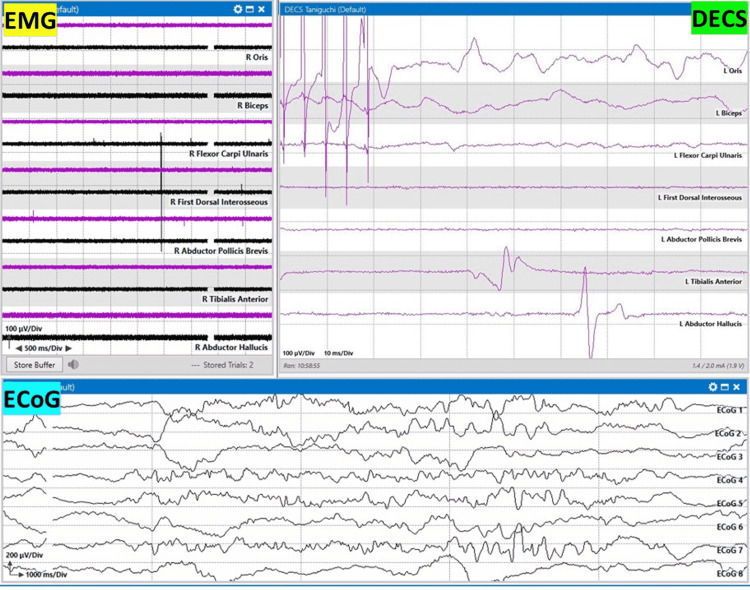
Taniguchi motor mapping method Taniguchi 320 Hz motor mapping evoked responses after monopolar handheld stimulation. Responses are recorded in the tibialis anterior (leg) and abductor hallucis (foot) muscles. Oris: orbicularis oris; biceps: biceps brachii; flexor carpi ulnaris, first dorsal interosseous, abductor pollicis brevis, tibialis anterior, and abductor hallucis muscles EMG: electromyography; DECS: Taniguchi direct electrical cortical stimulation; ECoG: electrocorticography

The Taniguchi method modifies the Penfield method by varying the pulse width and intensity and uses monopolar anodal direct cortical stimulation. For subcortical stimulation, the monopolar cathodal stimulation is used with this method. Using a train of five pulses, the duration of the pulse width should be 200-500 µs, with a stimulation frequency of 250-500 Hz. Stimulation intensity should be increased stepwise from 1 mA until a response is generated in the recording muscles until an after discharge occurs or until a maximum of 20 mA intensity is reached (Figures 4, 5). For subcortical stimulation, intensity should be decreased starting from 10 mA to determine the approximate distance from motor fibers, with 1 mA denoting approximately 1 mm from the corticospinal tract [9].

The recommended stimulation parameters for Penfield and Taniguchi methods are shown in Table 2.

**Table 2:** Recommended stimulation parameters for Penfield and Taniguchi methods Hz: Hertz; µs: microseconds; mA: milliamperes; s: seconds; ms: milliseconds

Specifications	Penfield	Taniguchi
Type of stimulator	Bipolar	Monopolar
Type of pulse (phase)	Biphasic or monophasic	Monophasic anodal or cathodal
Frequency	50 Hz	250-500 Hz
Pulse width	300-1,000 µs	500 µs
Intensity	2-20 mA	2-20 mA
Duration of stimulation	2-5 s	20 µs

Electrocorticography (ECoG) and electroencephalography (EEG)

Electroencephalography (EEG) baseline recordings using subdermal needle electrodes on the scalp are collected before the opening of the dura. Once the dura is opened, electrocorticography (ECoG) recordings are done with a subdural grid electrode placed near the area of stimulation in conjunction with DCS to monitor the possible development of any after discharges. The time base of the recording should be 500 ms/division with a gain of 200 and a sensitivity of 20-100 microvolts/division. The bandpass filter should be set at 1-70 Hz with no notch filter due to its tendency to attenuate seizure activity, decreasing the ability to detect after discharges (ADs). In the case of any after discharges caused by stimulation, ice cold saline (4°C) should be applied to the area to reduce seizure activity [5].

## Discussion

Wilder Graves Penfield is considered by many to be the father of modern epileptology and mapping of the brain. He dedicated himself to localizing their exact cortical positions to preserve sensory, motor, and speech areas. His work was based upon several scientists who preceded him, including Roberts Bartholow, who was the first to perform electrical stimulation of the cerebral cortex; Harvey Williams Cushing, who worked on mapping the homunculus in humans; and Otfrid Foerster, who first used ECoG intraoperatively and correlated the results of cortical electrical stimulation with deficits after resections. He published his representation of somatic motor and sensory areas in 1937, and the understanding of brain mapping has continued to evolve ever since. While the homunculus image reinforces the idea of the cortical localization of body areas, the cortical maps for motor stimulation of the different regions overlap significantly [1], necessitating precise mapping to avoid deficits.

While mapping the cortex is possible preoperatively, the brain may shift, and maps may be distorted due to lesions, edemas, or tumors. Thus, mapping of eloquent tissue is imperative. However, the risks may vary based on age, location of surgery, and comorbidity even with this. Additionally, simply avoiding impairment to the cortical surface is not sufficient as the possibility of infarcts and similar artery-related dysfunctions are a threat to the function of efferent corticospinal tracts at multiple levels across surgeries [11]. Consequently, motor deficits can occur if efferent nerve fibers from motor areas are impacted at any level. Combining these factors drove Taniguchi et al. to modify Penfield’s paradigm in 1993.

Without motor mapping, a wide variety of deficits may occur depending on the structures impacted, which would compromise a patient’s quality of life. Patients may see a reduction in muscle strength, slowing of movement, reduced coordination of voluntary movements, weakness with an impact on function, the need for an assistive device, or even paralysis where there is a complete loss of function and long-term contractures in the affected limb, a fate potentially sidestepped due to IONM’s capability in alarming the surgical team [4]. Examples include dropped shoulder, wrist, fingers, or foot; deviated, reduced, or missing flexion, extension, or rotational capability; restricted fine motor movement (apraxia); and numbness or pain. Motor damage may also impact the ability to move the mouth or eyes, causing a restriction or loss in the ability to speak and see effectively.

These deficits may be present because the patient suffered mechanical damage (irritation, compression, and severing), thermal damage (due to surgical instruments, electrodes, etc.), ischemia, or infarction impacting eloquent tissue during the surgery. In extreme cases, the damage may even lead to the patient’s death. Therefore, steps must be taken to ensure continuous intraoperative neurophysiologic monitoring (IONM) occurs and the creation of new deficits is actively avoided. The use of neuroimaging and SSEPs to localize and record from the motor cortex is insufficient. DECS is required to evoke a distal response and ensure that the motor pathways are intact. New or increased permanent deficits are less likely postoperative if both anatomical landmarks and DECS results are respected [12].

In addition, mapping helps maximize resection and reduce preoperative deficits to improve the patient’s quality of life. Ciric et al. found that 97% of patients had an improved or stable neurological status in gross or nearly gross total resections. In contrast, those with partial resections had a neurological morbidity rate of 40% [13]. Finally, motor mapping helps provide greater confidence to the surgeon that they are not impacting eloquent tissue when attempting a more extensive resection. Penfield and Taniguchi have developed different methods of DECS for mapping the motor cortex intraoperatively. While both share many parameters, their differences are significant and may have a critical impact on both the procedure and the result of surgery. At this time, both methods are proven to provide safe, helpful, and reliable resections near the central sulcus. Furthermore, both are complemented by subcortical motor mapping since tumors and other injuries may be present below the gray matter [2].

Of primary importance during any surgery is to minimize harm to the patient. We should consider the risk to the cortex due to DECS. Cortical stimulation aims to bring motoneurons from rest to their firing threshold by inducing excitatory postsynaptic potentials. Under anesthesia, this may require more stimulation to achieve the necessary temporal summation. The local polarization induced by Penfield’s bipolar stimulation may cause metal ions to move from the probe into the brain. This also alters the electrical sensitivity of the cortex to subsequent pulses (Niedermeyer’s electroencephalography). In addition, DECS with Penfield’s method is conducted over a longer time, resulting in a higher net charge. This increased stimulation intensity can affect spatial resolution due to the surface spread of the current. Taniguchi’s short-train stimulation is preferable as stimulation at smaller intensities reduces the likelihood of harm to the patient and increases the probability of correct localization. However, it is notable that at 250-400 Hz, the stimulation frequency in Taniguchi’s method rests above the “damage threshold” of 240 Hz, so there may still be a risk to the tissues [9].

Because high stimulus intensity is a risk factor, it should be monitored using ECoG to help track ADs and seizures [2,5]. As stated previously, it can counteract ADs by inducing local hypothermia with ice cold saline. Still, if an AD is not caught in time and results in a seizure, more profound anesthesia is required to prevent patient damage. Due to the propofol bolus needed, IONM can no longer continue, and therefore, any potential damage that may have been caught using those methods will no longer be preventable. Additionally, the seizure may still cause paralysis. Thus, it is vastly preferable to prevent ADs in the first place and then must catch and track them.

Shorter stimulation trains are less likely to generate this abnormal paroxysmal electrical activity while remaining efficient in localizing motor areas [14], which credence to the Taniguchi method’s benefit for safer motor mapping. Indeed, lower rates of seizure have been observed with successful motor and language mapping when using high-frequency (HF) monopolar cortical stimulation (MCS) as compared to low-frequency (LF) bipolar cortical stimulation (BCS) in patients with successful motor and language mapping [1]. Additionally, motor artifacts often interfere with micro-neurosurgeries and tumor resections, which are increased by longer-term stimulation, even without movement with seizure activity. Thus, HF stimulation is preferable for more precise readings [15], especially since movement is challenging to evaluate during anesthesia [9].

Other deficits may result from the direct impact of surgery on the motor pathways, whether through physical trauma, heat, ischemia, or other means. To avoid these tracts, it is not enough to preserve the primary motor cortex and the subcortical structures by which they are connected to the rest of the body. By design, bipolar stimulation causes current to flow from one surface node to another and thus is more effective for transverse axons in the cortex than longitudinal ones. In BCS, current density drops quickly with an increase in depth, and therefore, stimulation of the corticospinal tract (CST) is more difficult within the parameters of Penfield’s method. Monopolar stimulation does not draw the current back to the surface and therefore can directly excite vertically oriented pyramidal cells known to be of crucial importance to motor function due to their comprising most of the CST. Hence, the maintenance of these cells is indispensable. D waves are most effective for motor cortex identification under anesthesia [16], and stimulation of more than 150 Hz is required to effectively activate neurons 5 mm deep from the surface and elicit D waves [9].

Further, stimulation of the CST past the motor threshold (MT) increases the likelihood of a patient having postoperative motor deficits [2]. Suppose the patient is awake or under the recommended anesthetic protocol with no muscle relaxant on board. In that case, the MT is determined based on the approximately 1:1 ratio between the current (mA) required for excitation and the distance (mm) from the CST with monopolar short-train mapping [17]. An MT of 3-5 mA is the standard stop margin for resection. However, postoperative motor deficits from mechanical CST injury have returned to baseline within three months in patients with an MT between 1 and 20 mA.

Conversely, a wide range of distances has been recorded from bipolar stimulation sites to motor pathways, so finding an MT may be difficult. This is largely because BCS has less sensitivity and spatial resolution due to a smaller excitation area. Therefore, using a standard MT with BCS may be falsely reassuring and result in unexpected postoperative deficits [18].

Currently, subcortical mapping is carried out with BCS for mapping and MCS for monitoring descending pathways. However, if monopolar anodal stimulation is used for mapping, it eliminates the need for a bipolar probe. Thus, Taniguchi’s method would effectively achieve both subcortical motor mapping with less stimulation intensity and could continue to be used for intraoperative monitoring [5]. Furthermore, if MCS were to fail in mapping, BCS could always be used as a backup, but not vice versa. However, we would still need BCS for language mapping, so both may still be required based on where the surgery may impact eloquent tissue.

Patients undergoing cortical surgery often present with abnormal anatomy due to the pathology being treated, so the efficacy in mapping even with shifted brain structures is paramount. For example, BCS may fail to identify the motor cortex in a diseased brain [1]. In brains with premotor and parietal tumors, both HF and LF stimulation is effective. However, in M1 and M1 originating fibers, HF stimulation is far more effective, and LF also carries a high risk of negative mapping [18], suggesting that Taniguchi’s 250-500 Hz stimulation may be more effective in the precentral gyrus than Penfield’s 50 Hz stimulation paradigm. It is worth noting that sometimes negative mapping is sought to avoid the brain’s motor area. Still, a tumor or other anomaly may cause stimulation not to produce a response although it is in the correct area, leading to a false negative.

Taniguchi’s short-train method allows stimulation to be repeated quickly during tumor removal, thus providing more continuous monitoring. It also provides a better interpretation of waveforms’ latency, amplitude, and duration. With the Penfield method of stimulation, objectively analyzing factors such as latency and duration is more difficult as the excitation is summative over a more extended period [1]. While repeated DECS may appear to be a risk factor, using the tangential-radial triphasic (TRT) cortical model by adding a P25 peak directly on the cortical sensory generator reduces the need for higher stimulation parameters. It thus reduces the probability of postoperative deficits [7].

Considering all of this, it becomes clear that the Taniguchi model of monopolar high-frequency stimulation has many benefits over the Penfield model. Overall, there are fewer risks to the patient because of the reduced likelihood of ADs and seizures, more effective excitation and localization of cortical and subcortical motor tracts, and better mapping in patients with pathological neuroanatomy allow for more objective and precise temporal analysis. Additionally, bipolar stimulation remains a fallback option, while the opposite is not valid if mapping through monopolar stimulation fails.

Finally, and perhaps most importantly, expertise in mapping the motor cortex regardless of which method is used must be employed due to the high complexity of the task. A licensed IONM professional should be providing real-time supervision and accurate feedback based on data interpretation to minimize the risk of deficits [19]. In addition, as the mapping will vary from patient to patient based on their preoperative conditions and intraoperatively, the technician should communicate with the patient, surgeon, anesthesiologist, and nursing staff about patient history and procedures needed to reduce deficits. Therefore, a skilled technologist, neurophysiologist, remote physician, surgeon, multimodal mapping and monitoring, and thorough communication with everyone involved in the surgical procedure will produce the best results [19,20].

## Conclusions

Mapping of the motor cortex allows for faster and more accurate detection of the cortical and subcortical motor fibers, which minimizes deficits during surgical operations, especially in the hands of trained and experienced personnel. The standard Penfield stimulation method has a higher risk of causing after discharges, so the Taniguchi method, using higher stimulation frequency and shorter stimulation duration, provides a safer alternative to reduce seizure activity. We recommend using the Taniguchi method as the default for motor mapping in most cases for higher-risk surgeries. In cases where the Taniguchi method does not elicit responses or where the language cortex is involved, we should use the Penfield method instead for motor mapping.
